# How Barriers and Facilitators in the Community Environment Shape Opportunities for Healthy Aging With Disability

**DOI:** 10.1093/geroni/igaa057.2214

**Published:** 2020-12-16

**Authors:** Philippa Clarke, Martin Forchheimer, Lynn Charara, Ellen Wolgat, Michelle Meade, Denise Tate

**Affiliations:** University of Michigan, Ann Arbor, Michigan, United States

## Abstract

Due to advances in medical care and technology the average age of people living with early-acquired spinal cord injury (SCI) is increasing. Approximately 40% of adults with SCI are over age 65. However, the cumulative effects of living with a SCI for many years make aging with SCI different from those “aging into disability”. For example, unstable employment histories and the premature onset of secondary health conditions can create unique challenges for adults aging with SCI. Barriers and facilitators in the community environment play an important role for their ability to maintain health, engage in society, and participate in social roles. Data from a mixed methods study of ~200 adults (age 45+) aging with SCI, will be presented to demonstrate the impact of specific environmental barriers and facilitators and to stress the importance of understanding the complex dynamics of person-environment fit to fully support adults aging with and into disability. Part of a symposium sponsored by the Lifelong Disabilities Interest Group.

